# Homocystinuria patient and caregiver survey: experiences of diagnosis and patient satisfaction

**DOI:** 10.1186/s13023-021-01764-x

**Published:** 2021-03-10

**Authors:** T. Morrison, F. Bösch, M. A. Landolt, V. Kožich, M. Huemer, A. A. M. Morris

**Affiliations:** 1 HCU Network Australia, Baulkham Hills, Australia; 2 Division of Metabolism and Children’s Research Center, University Children’s Hospital Zurich, Zurich, Switzerland; 3 Division of Child and Adolescent Health Psychology, Department of Psychology, University of Zurich, Zurich, Switzerland; 4 Department of Psychosomatics and Psychiatry and Children’s Research Center, University Children’s Hospital Zurich, Zurich, Switzerland; 5 Department of Pediatrics and Inherited Metabolic Disorders, Charles University-First Faculty of Medicine, General University Hospital, Prague, Czech Republic; 6 Department of Paediatrics, Landeskrankenhaus Bregenz, Bregenz, Austria; 7 Division of Evolution and Genomic Sciences, Institute of Human Development, University of Manchester, Manchester, UK; 8 Willink Metabolic Unit, Manchester Centre for Genomic Medicine, Manchester University Hospitals NHS Foundation Trust, Manchester Academic Health Science Centre, Manchester, UK

**Keywords:** Cystathionine beta-synthase deficiency, Remethylation disorders, Patient support groups, Patient reported outcome, Delay in diagnosis

## Abstract

**Background:**

The main genetic causes of homocystinuria are cystathionine beta-synthase (CBS) deficiency and the remethylation defects. Many patients present in childhood but milder forms may present later in life. Some countries have newborn screening programs for the homocystinurias but these do not detect all patients.

**Results:**

HCU Network Australia is one of the very few support groups for patients with homocystinurias. Here we report the results of its survey of 143 patients and caregivers from 22 countries, evaluating current diagnostic pathways and management for the homocystinurias. Most (110) of the responses related to patients with CBS deficiency. The diagnosis was made by newborn screening in 20% of patients and in 50% of the others within 1 year of the initial symptom but in 12.5% it took over 15 years. The delay was attributed mainly to ignorance of the disease. Physicians need to learn to measure homocysteine concentrations in children with neurodevelopmental problems, and in patients with heterogeneous symptoms such as thromboembolism, dislocation of the optic lens, haemolytic uraemic syndrome, and psychiatric disease. Even when the diagnosis is made, the way it is communicated is sometimes poor. Early-onset CBS deficiency usually requires a low-protein diet with amino acid supplements. More than a third of the participants reported problems with the availability or cost of treatment. Only half of the patients always took their amino acid mixture. In contrast, good adherence to the protein restriction was reported in 98% but 80% said it was hard, time-consuming and caused unhappiness.

**Conclusions:**

There is often a long delay in diagnosing the homocystinurias unless this is achieved by newborn screening; this survey also highlights problems with the availability and cost of treatment and the palatability of protein substitutes.

## Background

The homocystinurias are rare genetic disorders affecting the metabolism of the non-proteinogenic amino acid, homocysteine. They are inherited as autosomal recessive traits. Homocysteine is formed during the catabolism of the amino acid, methionine. It can either be converted back to methionine by the remethylation pathway or converted to cystathionine and then to the amino acid, cysteine. Classical homocystinuria is caused by cystathionine beta-synthase (CBS) deficiency [1]. The worldwide incidence of CBS deficiency is about 1:100,000–1:200,000 [2], ranging from 1:1800 to 1:900,000 depending on ethnicity and method of case ascertainment.

The remethylation pathway depends on the coenzymes, methylcobalamin and methyltetrahydrofolate and the main remethylation disorders are defects of methyltetrahydrofolate recycling (methylenetetrahydrofolate reductase (MTHFR) deficiency) or cobalamin metabolism. The latter may only affect methylcobalamin or also affect adenosylcobalamin synthesis. All remethylation defects are very rare diseases. Population frequencies cannot even be estimated for cblC disease, the commonest remethylation defect, with several hundreds of published cases worldwide.

For each of these disorders, there is a range of severity and age of presentation. Severe remethylation disorders (RMD) present in infancy, primarily with neurological problems, such as acute encephalopathy (± seizures, apnoea or hydrocephalus) or severe developmental impairment [1, 3, 4]. Defects of cobalamin metabolism may also cause systemic problems, such as pancytopenia or haemolytic uraemic syndrome; patients may subsequently develop visual impairment due to retinopathy. Milder RMD may cause learning difficulties or present with ataxia, psychiatric problems or thromboembolism. The cobalamin-related RMD are all treated with large doses of hydroxocobalamin, which need to be given by intramuscular or subcutaneous injection. Patients with cobalamin defects are also treated with the drug, betaine, which converts homocysteine to methionine using an alternative pathway. Treatment improves survival and morbidity but cognitive and visual impairment persist in many severe, early onset cases. Betaine prevents neurocognitive deterioration if given early and is the drug of choice in patients with MTHFR deficiency, who should also be given mefolinate (methyltetrahydrofolate) as this is the only form of folic acid that crosses the blood brain barrier and it requires MTHFR for its synthesis [5].

Severe CBS deficiency presents in childhood with learning difficulties, dislocation of the optic lens, skeletal abnormalities and a predisposition to thromboembolism, whereas partial CBS deficiency typically presents in adults with thromboembolism [6, 7]. CBS deficiency is treatable: pyridoxal 5´-phosphate is a coenzyme for CBS and patients with milder forms of CBS deficiency usually respond to treatment (in terms of lowering tHcy to target levels or even close to the normal range) with pharmacological doses of its precursor pyridoxine. In contrast, patients with severe deficiency require treatment with a diet very low in natural protein and supplements of a methionine-free cystine-enriched amino acid mixture. The drug, betaine, can also help to lower homocysteine concentrations. All complications can be prevented with pyridoxine (in responsive patients) or dietary treatment, if it is started early and there is good compliance [6]. Currently, newborn screening programmes targeting at least one of the homocystinurias (most frequently CBS deficiency) are in place in several European countries such as the Czech Republic, Germany, Spain, the UK and Ireland; in several US states; in Qatar, Japan, Korea; and in Australia [8].

## Methods

### Online survey

HCU Network Australia is a support group established by TM in 2014. Initially, it aimed to support the Australian families with CBS deficiency but it has acquired international members with CBS deficiency and RMD as there are few other support groups specifically for these disorders. HCU Network Australia is not a member based organisation and as such the best way to describe its reach is based on its email/registry database. At the time of the survey there were 355 ‘members’/email contacts.

In 2018, an online survey was designed by TM using Survey Monkey software concerning the experiences of the homocystinuria community, primarily with respect to diagnosis and their satisfaction with current treatment. The design took account of previous publications on patient surveys [9–11]. The survey was reviewed and edited by VK and the president of HCU Network America, Margie McGlynn. The survey was trialled for clarity of questions by 2 Australian caregivers and 1 American adult patient.

A total of 59 questions were asked (see Additional file 1). These included:

- General demographic information (sex, age at diagnosis, ethnicity)
- Was detection by newborn screening?
- Tests done (e.g. tHcy level at diagnosis and most recent)
- Symptoms and age of onset for each
- Diagnostic delay and perceived reasons
- Patient satisfaction with diagnostic process
- Treatment (type, frequency and mode of delivery)
- Patient satisfaction with treatment regime (e.g. low protein diet, amino acid mixture, medication)
- Satisfaction with medical care

There were opportunities for free comments (including about diagnosis and current treatment).

The 355 existing email contacts registered with HCU Network Australia at the time were contacted and invited to complete the survey online. The survey was placed on the HCU Network Australia website (www.HCUNetworkAustralia.org.au) and visitors to the website were prompted to complete the survey. Information about the survey was also presented on the European Registry for Homocystinurias and Methylation Defects (E-HOD) project website (www.e-hod.org) and circulated on the social media channels of HCU Network Australia, of the partnering patient organisation based in the US, HCU Network America, and of Cambrooke, an international medical foods company. In the Czech Republic the survey questions were translated into Czech with help from the patient organization NSPKU (Národní sdružení PKU a jiných DMP, z.s.) and patients and/or carers were invited to fill in appropriate fields in the on-line survey in English or Czech. The invitation to participate was distributed via announcement by NSPKU as well as by mailing to 37 patients followed in the General University Hospital. Anonymous entries in Czech were translated back to English by VK and deposited into the survey database. The survey was open from May 2018 to April 2019.

The study was initiated and carried out by patient organization HCU Network Australia, data were anonymous and no ethics committee approval was sought. The “Kantonale Ethikkommission Zürich” raised no concern regarding statistical analysis of the anonymised survey data.

### Data cleaning

Double entries were detected from the IP addresses of the participants. In each case the most complete version was included. Two patients with methionine adenosyltransferase I/III deficiency were excluded, as the clinical course is distinct from the homocystinurias. Extreme, biochemically implausible outliers (e.g. total Hcy values exceeding 1000 µmol/l, probably confused with methionine) as well as illogical answers (e.g. newborn screening not performed but diagnosis made by newborn screening) were excluded and handled as missing data.

### Statistical methods

Statistical analyses were carried out with the statistical software package SPSS, version 24 for Windows [12]. For all tests a predefined significance level of *p* < 0.05 was set. Chi-square tests were conducted to assess differences between diagnosis subgroups (CBS and RMD) regarding reported symptoms and current treatment regimen. Chi-square tests were also used to compare the psychological burden associated with different treatments (low protein diet, intake of amino acid mixtures, medication). A Fisher's exact test was used if the number in any group was less than 5. Dependent t-tests were conducted to examine differences between most recent tHcy concentrations and tHcy concentrations prior to treatment. Two-tailed Pearson correlations were conducted to examine the relationship between age and diagnosis delay. Multiple linear regression analyses were conducted to determine predictors of respondents’ satisfaction with treatment and medical care. The a priori defined predictors [6, 13, 14] were patient age, patient gender, mode (newborn screening or clinically ascertained) and type of diagnosis (CBS, RMD), self- vs. proxy reporting, and diagnostic delay.

## Results

Between May 2018 and April 2019, the survey was completed by 143 participants. Patient characteristics are summarised in Table 1.

**Table 1:** Patient characteristics

	N	Median (IQR); mean ± SD
**Patient age at the time of the survey**		
Age of all patients	138	18 years (6–38); 22.7 ± 18
Number of paediatric patients (≤ 18 years)	69	
Age of patients identified by newborn screening	29	4 years (2–22); 11.5 ± 13.2
Age of clinically ascertained patients	76	21 years (10–38); 24.8 ± 17.3
	N	%
**Participants**	143	
Male	60	42
Female	81	56.6
No information provided	2	1.4
**Informants**		
Caregivers	82	57.3
Adult patients (> 18 years)	55	38.5
Adolescent patients (< 18 years)	2	1.4
Health professionals	3	2.1
No information provided	1	0.7
**Countries of origin**	141	
USA	46	32.6
Czech Republic	20	14.2
United Kingdom	19	13.5
Australia	17	12.1
Ireland	8	5.7
Germany	6	4.3
Other European countries	9	6.5
South America	4	2.8
Asia	4	2.8
Canada	4	2.8
Africa	2	1.4
New Zealand	2	1.4
**Diagnosis**	142	
* Cystathionine beta-synthase (CBS) deficiency*	110	77.5
Pyridoxine nonresponsive	62	
* Diagnosed by newborn screening*	21	
Partially pyridoxine responsive	8	
* Diagnosed by newborn screening*	1	
Pyridoxine responsive	28	
* Diagnosed by newborn screening*	–	
Unsure of responsiveness	12	
* Diagnosed by newborn screening*	2	
*Remethylation defects (RMD)*	24	16.9
cblC	10	
* Diagnosed by newborn screening*	4	
MTHFR deficiency	7	
*Diagnosed by newborn screening*	–	
cblE	3	
*Diagnosed by newborn screening*	–	
cblG	3	
*Diagnosed by newborn screening*	–	
cblF	1-	
*Diagnosed by newborn screening*	–	
Unsure of diagnosis	8	5.6
*Diagnosed by newborn screening*	1	

Cbl, cobalamin; IQR, interquartile range; MTHFR, methylenetetrahydrofolate reductase; SD, standard deviation

### Establishing the diagnosis

Median age at diagnosis in clinically ascertained patients was 3 years with a wide range between < 1 and 60 years. Newborn screening for homocystinuria was reported to have been undertaken in 43 (30%) patients and led to the diagnosis in 29 (67%) of these patients (Table 1). The remaining 14 (33%) patients who had a newborn screening but were not detected had MTHFR deficiency (2), cblC disease (1), partially pyridoxine responsive (2), and pyridoxine non-responsive (9) CBS deficiency. The diagnoses may not actually have been missed, as it is unknown which screening tests were undertaken in these patients in the newborn period.

In addition to those detected by newborn screening, 14 patients (10%) were diagnosed in the first year (4 non-pyridoxine responsive CBS deficiency; 1 unsure regarding responsiveness; 3 cblC, 1 cblG, 1 cblE defect; 2 MTHFR deficiency; 2 could not name the diagnosis). Distribution of diagnostic delay did not differ significantly between CBS and RMD and between subgroups of CBS according to pyridoxine responsiveness.

Plasma total homocysteine concentrations (tHcy) were significantly higher before compared to during treatment (i.e. at the time of the survey) (Table 2).

**Table 2:** Plasma total homocysteine concentrations (tHcy) before and during treatment (i.e. at the time of the survey)

	tHcy (µmol/l) before treatment median (range) [n]	tHcy (µmol/l) at survey median (range) [n]	Statistical significance of difference [pairwise comparisons n] *t-values*
All CBS-deficient patients	257 (50–640) [44]	50 (6–310) [61]	P < 0.001 [37]
			*10.804*
Pyridoxine nonresponsive	200 (50–640) [20]	52 (6–310) [34]	P < 0.001 [15]
			*6.837*
Partially pyridoxine responsive	300 (99–412) [7]	26 (13–120) [8]	P = 0.001 [7]
			*5.976*
Pyridoxine responsive	257 (110–600) [12]	59 (11–180) [14]	P < 0.001 [10]
			*6.166*
RMD patients	145 (56–482) [11]	42 (7–95) [14]	P = 0.006 [11]
			*3.433*

**Fig. 1:**
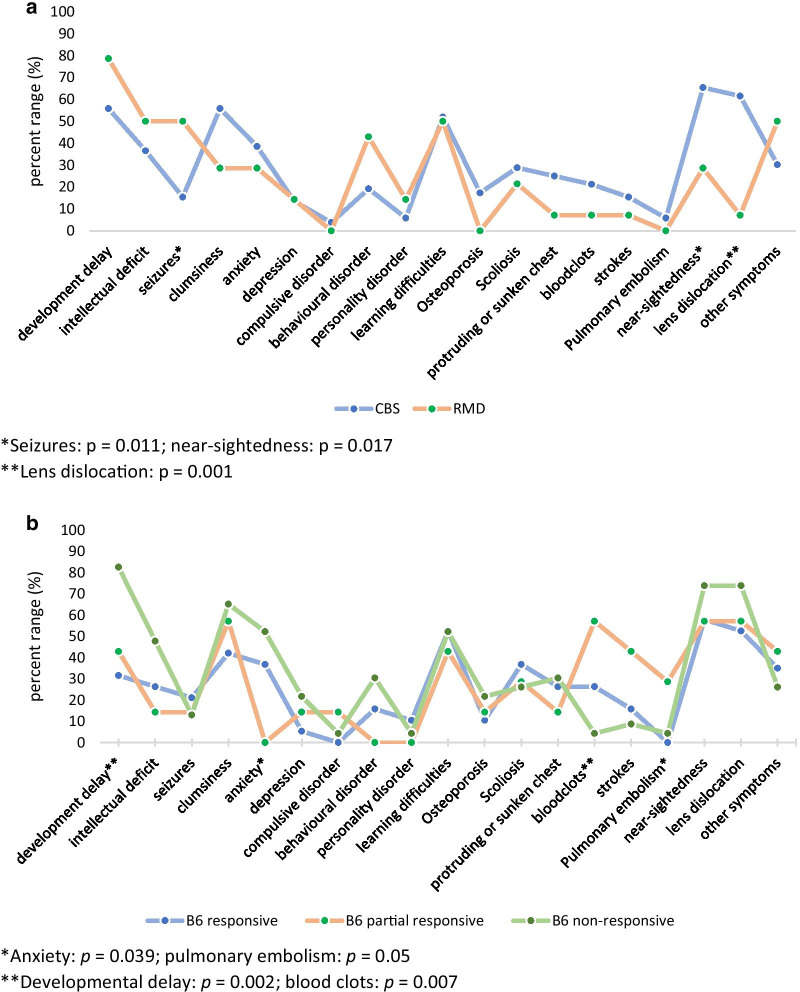
**a** Percentage of self-/proxy-reported symptoms in clinically ascertained patients with CBS deficiency (n = 52) and RMD (n = 14). **b** Percentage of self-/proxy-reported symptoms in clinically ascertained pyridoxine responsive (n = 19), partially responsive (n = 7) and nonresponsive (n = 23) CBS patients

Symptoms were reported for 66 clinically ascertained patients (46.2%) (Fig. 1). The most common symptoms in infancy and childhood were developmental delay, learning difficulties and behaviour problems, reaching a peak at 2–4 years of age. Other common symptoms in childhood were clumsiness (mostly at 1–4 years) and myopia/lens dislocation (most often at 3–4 years, though there was more than 1 case per year till 8 years). Significant differences of symptom frequencies between clinically ascertained CBS and RMD patients were detected for lens dislocation (*p* = 0.001) and myopia (“near-sightedness”; *p* = 0.017), which occurred almost exclusively in CBS deficiency, and seizures (*p* = 0.011), which were more frequent in RMD patients.

Clinically ascertained pyridoxine nonresponsive CBS patients reported significantly more frequently developmental delay (*p* = 0.002) and anxiety (*p* = 0.039) while responsive CBS patients had more thromboses (“blood clots”; *p* = 0.007) and pulmonary embolism (*p* = 0.05).

For 71 patients, the ages of the onset of symptoms and of diagnosis were both reported. Patient age at first symptoms correlated significantly with diagnostic delay (*r* = 0.246, *p* < 0.05). In clinically ascertained patients the diagnosis was made within a year of the initial symptom in 50% of patients, but in 12.5% it took > 15 years. The delay in diagnosis was largely attributed to doctors’ ignorance of the disease or to organisational problems. Forty-five of the patients not identified by newborn screening (including 3 patients diagnosed within the first year of life) were initially given an incorrect diagnosis, such as Marfan syndrome, dyspraxia, developmental delay, behavioural or psychiatric problems, or autoimmune disease. Metabolic specialists (n = 24), ophthalmologists (n = 18), paediatricians (n = 8), neurologists (n = 6) or geneticists (n = 5) were the physicians who most frequently raised the possibility of a homocystinuria.

**Fig. 2:**
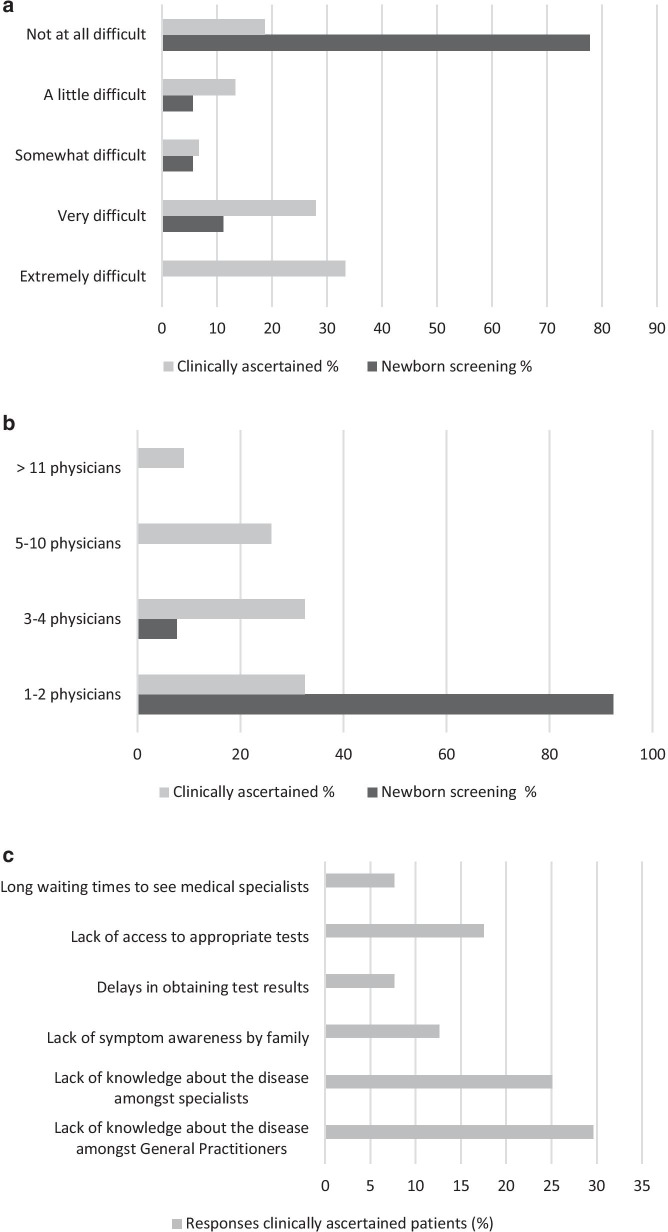
The perceived difficulty of diagnosis for patients identified by newborn screening (n = 18) or ascertained clinically (n = 75) (**a**), the number of physicians seen prior to diagnosis for patients identified by newborn screening (n = 13) or ascertained clinically (n = 77) (**b**) and the perceived reasons for diagnostic delay (multiple answers possible) in clinically ascertained patients (**c**)

The perceived difficulty of diagnosis, the number of physicians seen prior to diagnosis for patients identified by newborn screening or ascertained clinically and the perceived reasons for diagnostic delay are depicted in Fig. 2.

### Communication of diagnosis

The diagnosis was given in person to 66% of patients/parents, by telephone to 23% and in a letter to 1%. Only 26% of patients were offered psychological support. Of 102 respondents, 62% percent were satisfied with the way the diagnosis was delivered, 18% were neither satisfied nor dissatisfied but 17% thought it was unsatisfactory. Twenty-four percent of respondents felt they had been given insufficient information about the disease. Almost all respondents (98%) said they were interested in being kept informed of current research and clinical trials in the disease. Most respondents (90%) were extremely, highly or somewhat interested in joining support groups but only 21% were given information about these at the time of diagnosis and only 50% had found a relevant group in their country.

Free comments by participants showed that dissatisfaction with communication of the diagnosis was mainly related to the perception of insufficient information provided, inappropriate communication behaviour and limited support from medical staff: “I would have preferred to have a psychological support”; “they didn't even ask if my husband wanted to be there”. Some comments related to negative feelings elicited by the diagnosis: “shock, guilt, regret, grief, despair, hopeless”. Positive comments mostly related to finally having achieved a diagnosis (now I know what I have/my child has), or appreciated medical staff showing “kindness”, “support”, “concern” and “empathy” that made families “feel like a priority”.

### Current support and medical care

Information about current medical care was reported by 99 participants. Eighty-nine of these said they attended a specialist clinic; 82 saw a metabolic specialist, 21 a geneticist, 12 a haematologist, 10 a paediatrician, 9 a neurologist and 17 a general practitioner—obviously, some saw more than one doctor but 2 reported having no health professional who managed their homocystinuria.

Care was considered good or excellent by 67% of respondents, average or fair by 29% and only 4% considered it poor. Nevertheless, 20% of respondents reported some difficulty talking to medical staff at clinics and a similar number reported difficulty getting an answer to questions between clinics.

### Treatment

One hundred and five people gave information about treatment. Ten patients (9%) with CBS deficiency received parenteral vitamin B12, which is not recommended as a disease-specific treatment but may have been used to supplement nutritional deficiencies.

**Fig. 3:**
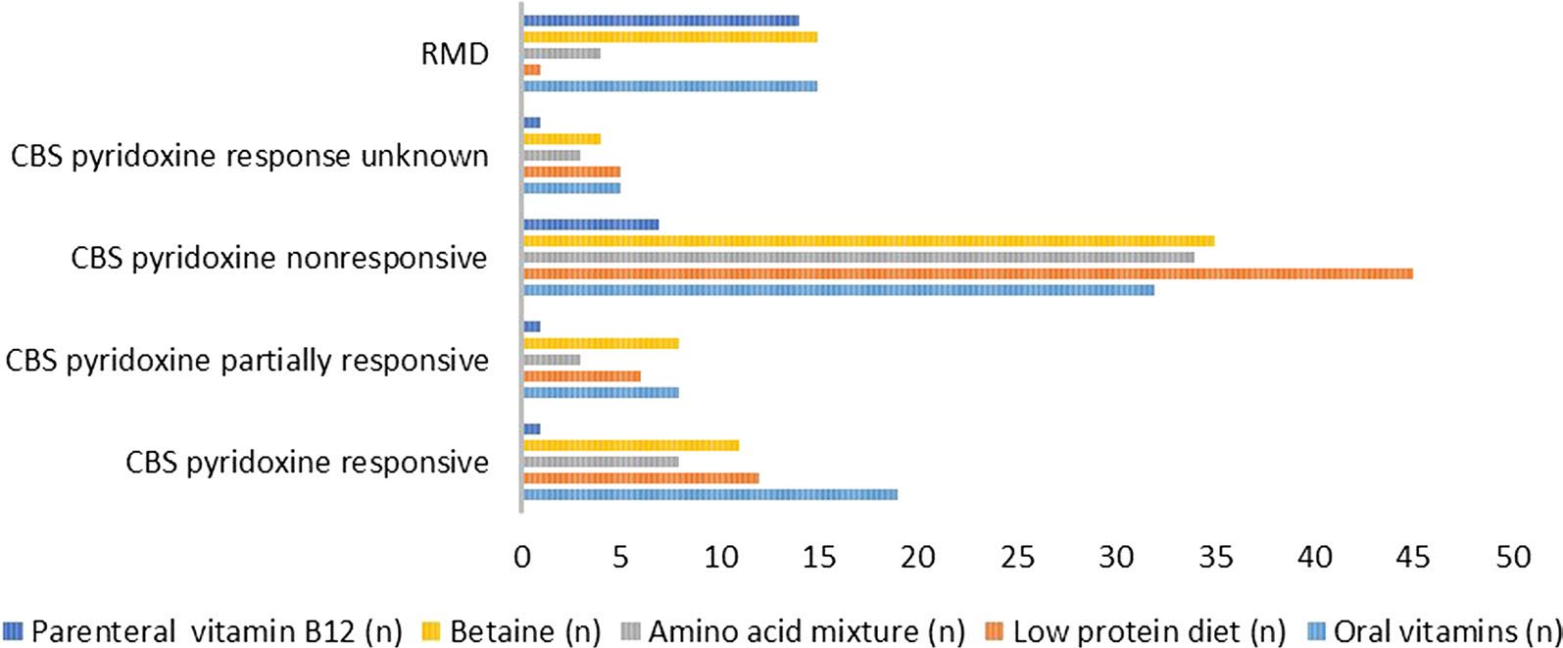
Treatment in CBS responsiveness subgroups and patients with RMD

As expected, low protein diets (*p* = 0.001), methionine-free amino acid supplements (p = 0.01), and betaine (*p* < 0.05) were all used by a significantly higher proportion of pyridoxine nonresponsive than responsive CBS-deficient patients (Fig. 3). One RMD patient was said to be on a low-protein diet, which is not recommended; 4 were taking amino acid mixtures.

### Treatment-related problems and patient satisfaction

Nearly forty percent of participants were extremely (11.1%) or very (27.3%) satisfied with the treatment but 13.1% of patients were very dissatisfied and almost fifty percent considered treatment only partly satisfactory (somewhat = 40.4%; a little = 8.1%). The psychological burden of low protein diet, intake of amino acid mixtures and medication is shown in detail in Table 3.

**Table 3:** Psychological burden of low protein diet, and intake of amino acid mixtures and medication

	Never	Yes	A little of the time	Sometimes	Most of the time	Always
*In the past seven days did you/was it…*						
Argue with your child about intake of amino acid mixture? (n = 47)*	13	34	6	14	4	3
	28%	72%	18%	41%	12%	9%
Hard to take amino acids? (n = 48)	13	35	14	13	4	4
	27%	73%				11%
Hard to engage in activities due to intake of amino acid mixture? (n = 58)	28	30	12	12	4	2
	48%	52%	40%	40%	13%	7%
Argue with your child about low protein diet? (n = 39)	17	22	11	11	–	–
	44%	56%	50%	50%		
Hard to follow low protein diet? (n = 65)	12	53	18	22	10	3
	19%	81%	34%	41%	19%	6%
Hard to engage in activities due to low protein diet? (n = 53)	23	30	16	9	–	5
	43%	57%	53%	30%		17%
Did patient follow the low protein diet as directed? (n = 45)	1	44	2	9	–	33
	2%	98%	4%	21%		75%
Feel unhappy about diet? (n = 61)	11	50	15	19	11	5
	18%	82%	30%	38%	22%	10%
Enjoy eating when following diet? (n = 52)	1	51	8	9	–	34
	2%	98%	16%	18%		66%
Want to eat what others eat? (n = 53)	11	42	8	17	–	17
	21%	79%	19%	40.5%		40.5%
Preparing low-protein meals time consuming? (n = 48)	10	38	14	11	–	13
	21%	79%	37%	29%		34%
Annoying/challenging to weigh or estimate protein content? (n = 49)	13	36	12	12	–	12
	27%	73%	33.3%	33.3%		33.3%
Hard to take oral/parenteral medication? (n = 74)	43	31	11	13	–	7
	58%	42%	35%	42%		23%

^*^7 participants chose no category but explained verbally that there were arguments with the child about intake of the amino acid mixture

Most participants (n = 67) reported no problems with the availability or affordability of drugs and vitamins but 37 reported problems related to availability (“struggle to contact suppliers. Always out of stock”; “not available in my country”), financial burdens (“very expensive and the insurance does not cover”) or cumbersome administrative and logistic processes (“My insurance doesn't cover betaine and it takes months at a time to get it covered for a month's supply. By the time I am covered my husband usually switches jobs (he does contract work) so we have a different insurance provider and then they deny coverage of my medication and we have to go through the same long process over again to get the drug covered. It's a vicious cycle”).

The financial burden on some patients was illustrated by the respondents’ estimate of the monthly additional costs of medications or vitamins to their family to a mean of 231 € (median 46; range 0 to 4495€). Additional monthly costs for specialised food accumulated to 154 € (median 75; range 0 to 1798 €).

Pairwise comparisons showed that carers reported significantly more arguments with their child about taking the amino acid mixture than about the protein restriction (*p* = 0.001). The need for amino acid mixtures also affected participation in activities more often than the protein restriction (*p* < 0.01). Patients who were on oral/parenteral medication and a low protein diet found it harder to take their medication (p < 0.01) than to adhere to the protein restriction.

Ninety-eight respondents said they/their child had adhered to the diet in the last seven days. Fifty percent of respondents (n = 23) reported always taking their amino acids but five (11%) reported missing it at least once per day.

### Patient satisfaction with medical care

The regression model including patient age, gender, type of diagnosis (CBS, RMD), diagnostic mode (diagnosed by newborn screening/clinical ascertainment), source of report (self/proxy) and diagnostic delay as predictors and respondents’ satisfaction with treatment and medical care as an outcome was significant F(6,54) = 2.937, p = 0.016, adjusted R2 = 0.177). Patients’ age was a significant predictor (standardized β = 0.37, p = 0.039), older participants generally being more satisfied with their treatment and medical care. Additionally, diagnosis by newborn screening predicted significantly higher treatment satisfaction (standardized β = 0.344, p = 0.013).

## Discussion

Physicians, healthcare providers and companies producing medication, special food or amino acid mixtures all have much to learn from this survey, which is exceptional in being designed and undertaken by patient representatives without the influence of medical professionals. It can, therefore, provide valuable insight into the problems faced by families with homocystinuria. Most of the subjects had CBS deficiency with small numbers of RMD patients.

Patients and carers with these rare diseases clearly feel that diagnosis and treatment are significantly delayed by the ignorance of doctors and sometimes a shortage of diagnostic facilities and experts. Similar problems are experienced by patients with other rare diseases [15]. Clearly, one cannot expect general practitioners, physicians and paediatricians to be experts on all rare diseases. Unfortunately, the ‘diagnostic odyssey’ is particularly marked with the homocystinurias because most of the clinical features are non-specific, such as learning difficulties and clumsiness in children and thromboembolism in adults. The patient or family’s frustration is compounded when the diagnosis is eventually made (following irreversible damage) and they learn that the condition is treatable. Metabolic specialists must teach mainstream specialist colleagues to have a low threshold for measuring plasma total homocysteine in patients with thromboembolism and children with learning difficulties. It should also be measured in all patients with dislocation of the optic lens, the most specific feature for CBS deficiency: several patients in this survey were initially misdiagnosed as Marfan syndrome, as in previous series [16].

The diagnosis was made within a year of the first symptoms in half of the clinically ascertained patients in this survey but it was delayed by more than 15 years in 12.5% patients. The patient’s age at first presentation correlated with the diagnostic delay. Long diagnostic delays have been found in previous series [16] and the delay has sometimes correlated with age [7, 13]. This reflects the particularly non-specific features in adult onset patients, who are generally pyridoxine-responsive. The data are compatible with those from the European Registry for Homocystinurias and Methylation Defects (E-HOD), which showed a median diagnostic delay of 1.6 years for pyridoxine-nonresponsive patients with CBS deficiency, increasing to 2.4 years in partial responders, 5.1 years in full and 14 years in extreme responders requiring only low doses of pyridoxine [7]. The RMD in this series were diagnosed in early childhood, with a median delay of 24 days, suggesting they were mostly severe, early-onset cases.

Newborn screening is well suited to treatable diseases whose clinical diagnosis is often delayed. Approximately one quarter of the patients in this survey had been detected by newborn screening and reported higher satisfaction with the diagnostic procedure and with treatment and medical care than those detected clinically. These numbers are relatively high since few countries undertake newborn screening for CBS deficiency and even fewer for RMDs; moreover, most of these have only introduced screening recently [8]. Reasons why countries do not screen for homocystinurias may include their rarity, high false positive rate if second tier tHcy is not measured and high false negative rate for pyridoxine-responsive CBS deficiency and some RMD [8]. In this survey, 29 patients were ascertained by newborn screening while 14 patients who underwent newborn screening were not detected in the newborn period and were ascertained clinically; in the latter group of patients, it is unclear whether at the time of blood collection the respective screening programmes targeted the relevant form of homocystinurias.

The majority of patients thought that the diagnosis had been delivered in a satisfactory way but the survey shows there is still room for physicians to improve their communication skills and to provide more information about the disease. Some respondents were unable to name their disease or to provide information about parameters critical for clinical decision making such as their plasma homocysteine values. Standardised, valid education materials for patients with inborn metabolic diseases have recently been developed [17] and are helpful for medical professionals and patients. Written information about CBS deficiency and RMDs is available in most European languages on http://www.e-hod.org. Sadly, it comes as no surprise to find that few families were offered support from a psychologist following diagnosis as this resource has limited availability in many healthcare systems. Patients should, however, be informed about local metabolic support groups or international ones, such as HCU Network Australia, HCU Network America and cblC Onlus, Italy. In this survey, few were informed about support groups though most are keen to join and want to be kept informed of research and clinical trials.

Additional problems for patients and carers include the cumbersome procedures for obtaining medications, amino acid supplements and other dietary products and—in some countries—the high cost of these. For patients on dietary treatment, taking the amino acid mixtures is reported to cause more problems than the dietary protein restriction. The amino acid mixtures available for CBS deficiency are less palatable than those for other disorders, such as phenylketonuria; this is related to the amino acid composition, such as the need for cystine, but it is clearly something for the medical food industry to work on. The diet does, however, cause significant stress: many patients do not enjoy eating and resent being unable to eat like others; parents complain about frequent discussions about food with their affected children. It has recently been shown that patients with phenylketonuria and acute intoxication-type inborn errors of metabolism who should adhere to a protein-restricted diet have significantly impaired health-related quality of life [18]. All of these factors contribute to the unmet need for psychological, financial and social support in patients and families with homocystinuria.

The survey suggests that treatment of the homocystinurias is not always in line with current recommendations [3, 6]. One patient with a RMD was on protein restriction and 4 were said to receive amino acid mixtures. Protein restriction is not recommended in RMD. It was not specified whether amino acid intake was methionine (which would be appropriate treatment) or a general amino acid mixture (which would not). Ten patients with CBS deficiency were on parenteral vitamin B12 treatment, though this should only be necessary in RMD. It is possible that some patients with CBS deficiency had been vitamin B12 deficient and struggled with oral supplements. Alternatively, it may be that some patients were receiving the (painful) injections unnecessarily. Approximately two thirds of the pyridoxine-nonresponsive CBS deficient patients also took oral vitamins. Routine folic acid supplementation has been recommended for CBS deficiency as it is cheap and easy to take and it is important to avoid deficiency but oral pyridoxine is only indicated in responsive patients. Standardised testing of pyridoxine responsiveness in CBS deficiency has recently been recommended and would be helpful to tailor treatment more precisely.

A low-methionine diet is recommended for pyridoxine-nonresponsive CBS deficient patients, achieved by severe protein restriction and methionine-free amino acid supplements. A quarter (27%) of these patients were on a normal diet. They may have been late diagnosed patients in whom diet is hard to introduce—resulting in sub-optimal treatment—or patients who have achieved homocysteine levels in the target range by taking betaine. Twenty patients with CBS deficiency were on a low-protein diet without an amino acid mixture. This may have been due to difficulty persuading some patients to take supplements whose taste is not particularly pleasant. Sadly, in a number of cases the reason was probably unavailability of suitable amino acid mixtures, either because the patient cannot afford them or due to supply problems in their country. In these circumstances, treatment with a milder dietary protein restriction (minimum safe intake) and betaine may be the best that can be achieved. These patients require close monitoring of their nutritional status and dietary intake.

While this study is unique with regard to providing information from patients and caregivers, several limitations, mainly related to selection bias, need to be taken into account. The selection of topics the survey addresses was driven by individual experiences. Therefore, some aspects (e.g. the pathway to diagnosis) are covered in greater detail than others (biochemical values) and the results of this survey may not be completely representative. The majority of patients have a form of classical homocystinuria and very few individuals with RMD are included. The survey was distributed via patient support groups and the internet, which might have skewed participation towards people involved in such groups and competent with internet-based surveys. The survey was in English with only a Czech translation available, which probably led to overrepresentation of English-speaking countries and people with above average education from other countries. There may also have been some inaccuracy in the reporting of symptoms and biochemical values.

## Conclusions

This survey was completed by an international group of carers and patients, most of whom had CBS deficiency with a smaller number of RMDs. Approximately one quarter of the patients had been detected by newborn screening. For many of the other patients, there was a long delay in diagnosis. Most of the patients are now under a metabolic specialist. Few were informed about support groups though most are keen to join these and receive more information about the disease. There is room for improvement of communication between medical professionals and patients/caregivers. Forty percent of participants were satisfied with the treatment but about 20% of patients were very dissatisfied and 40% considered treatment only partly satisfactory. This may in part reflect the diagnoses. Treatment is easy and effective for pyridoxine-responsive CBS-deficient patients but dietary treatment is difficult for other CBS-deficient patients. Treatment is not very effective for patients with RMDs and patients with cobalamin defects may dislike their hydroxocobalamin injections.

## Supplementary Information

**Additional file 1**. The Survey Monkey questionnaire.

## Data Availability

The datasets used and/or analysed during the current study are available from the corresponding author on reasonable request.

## References

[1] Baumgartner MR, Fowler B, Blau N, Duran M, Gibson KM, Dionisi Vici C (2014). Vitamin B12 disorders. Physician's guide to the diagnosis, treatment, and follow-up of inherited metabolic diseases.

[2] Moorthie S, Cameron L, Sagoo GS (2014). Systematic review and meta-analysis to estimate the birth prevalence of five inherited metabolic diseases. J Inherit Metab Dis.

[3] Huemer M, Diodato D, Schwahn B (2017). Guidelines for diagnosis and management of the cobalamin-related remethylation disorders cblC, cblD, cblE, cblF, cblG, cblJ and MTHFR deficiency. J Inherit Metab Dis.

[4] Scaglia F, Blau N, Blau N, Duran M, Gibson KM, Dionisi Vici C (2014). Disorders of folate metabolism and transport. Physician's guide to the diagnosis, treatment, and follow-up of inherited metabolic diseases.

[5] Knowles L, Morris AAM, Walter JH (2016). Treatment with mefolinate (5-methyltetrahydrofolate), but not folic acid or folinic acid, leads to measurable 5-methyltetrahydrofolate in cerebrospinal fluid in methylenetetrahydrofolate reductase deficiency. JIMD Rep.

[6] Morris AAM, Kožich V, Santra S (2017). Guidelines for the diagnosis and management of cystathionine beta-synthase deficiency. J Inherit Metab Dis.

[7] Kožich V, Sokolová J, Morris AAM (2020). Cystathionine β-synthase deficiency in the E-HOD registry-part I: pyridoxine responsiveness as a determinant of biochemical and clinical phenotype at diagnosis. J Inherit Metab Dis..

[8] Keller R, Chrastina P, Pavlíková M (2019). Newborn screening for homocystinurias: recent recommendations versus current practice. J Inherit Metab Dis.

[9] Hooper M, Hudson P, Porter F, McCaddon A (2014). Patient journeys: diagnosis and treatment of pernicious anaemia. Br J Nurs.

[10] Anderson M, Elliott EJ, Zurynski YA (2013). Australian families living with rare disease: experiences of diagnosis, health services use and needs for psychosocial support. Orphanet J Rare Dis.

[11] Ford S, O'Driscoll M, MacDonald A (2018). Living with Phenylketonuria: Lessons from the PKU community. Mol Genet Metab Rep.

[12] IBM Corp. IBM SPSS Statistics for Windows. 2016.

[13] Huemer M, Diodato D, Martinelli D (2019). Phenotype, treatment practice and outcome in the cobalamin-dependent remethylation disorders and MTHFR deficiency: data from the E-HOD registry. J Inherit Metab Dis.

[14] Molema F, Gleich F, Burgard P (2019). Evaluation of dietary treatment and amino acid supplementation in organic acidurias and urea-cycle disorders: on the basis of information from a European multicenter registry. J Inherit Metab Dis.

[15] Blöß S, Klemann C, Rother AK (2017). Diagnostic needs for rare diseases and shared prediagnostic phenomena: results of a German-wide expert Delphi survey. PLoS ONE.

[16] Cruysberg JR, Boers GH, Trijbels JM, Deutman AF (1996). Delay in diagnosis of homocystinuria: retrospective study of consecutive patients. BMJ.

[17] Zeltner NA, Welsink-Karssies MM, Landolt MA (2019). Reducing complexity: explaining inborn errors of metabolism and their treatment to children and adolescents. Orphanet J Rare Dis..

[18] Bösch F, Landolt MA, Baumgartner MR (2020). Health-related quality of life in paediatric patients with intoxication-type inborn errors of metabolism: analysis of an international data set. J Inherit Metab Dis..

