# Supplementary material for: Homocystinuria patient and caregiver survey: experiences of diagnosis and patient satisfaction
Source: Orphanet J Rare Dis. 2021 Mar 10;16:124. doi: 10.1186/s13023-021-01764-x (PMC7945666; doi:10.1186/s13023-021-01764-x)
Supplement: Supplementary file 1 — Additional file 1. The Survey Monkey questionnaire. [file 13023_2021_1764_MOESM1_ESM.pdf]

Welcome to the Homocystinuria Patient and Caregiver Survey

Who is HCU Network Australia?

HCU Network Australia is a Health Promotion Charity established in 2014 for individuals affected by homocystinuria and their families. Our aim is to connect and support HCU individuals and their families, provide information and education to persons impacted by the disorder and encourage and support medical expertise in the area.
You should answer this survey if you, or a family member, have been diagnosed with one of these disorders. If you are responding as a parent/caregiver and have MORE THAN ONE child with homocystinuria, please submit a survey response for EACH CHILD.

There is currently limited evidence showing the considerable length of time taken to reach a diagnosis of homocystinuria and levels of patient satisfaction regarding access to information, treatment options and medical care. This survey is important to highlight the current situation and has been prepared in consultation with a Key Opinion Leader (KOL) in Europe together with input from HCU Network America, to ensure the information gathered can be used globally to help support improved care for all homocystinuria patients.

We ask no matter what your experience, good or bad, you complete the survey.

The survey will be anonymous and your identity will not be known. You can refrain from answering any question that you feel could lead to your identity.

The aggregate results of the survey will be published.

If you have any questions please contact: Tara Morrison (Director, HCU Network Australia) at tara@hcunetworkaustralia.org.au
The deadline for completion of this survey is Sunday 1st February 2019.
The survey will take approximately 15 minutes to complete.

About the affected individual

1. Who is answering the questions in this survey?
   - Affected adult (18 years and above)
   - Affected adolescent (12 - 17 years)
   - Caregiver
   - Other

If 'other' please specify. ____

2. Are you or your child male or female?
   - Male
   - Female

3. What is your or your child's current age? ______________________________

4. In what country do you or your child live? ______________________________

5. What type of homocystinuria do you or your child have?
   - Classicial homocystinuria (HCU) / cysthathionine beta-synthase (CBS) deficiency
   - Methylenetetrahydrofolate reductase (MTHFR) deficiency
   - Cobalamin C (CblC) disorder
   - Cobalamin D (CblD) disorder
   - Methionine synthase reductase (CblE) deficiency
   - Cobalamin F (CblF) disorder
   - Cobalamin J (CblJ) disorder
   - Unsure
   - Other

If 'other' please specify. ____

6. If you answered classical homocystinuria above please specify the type below.
   - Vitamin B6 (pyridoxine) responsive
   - Vitamin B6 non-responsive
   - Vitamin B6 partial responsive
   - Unsure

Please provide any additional comments. ____

Experience of diagnosis

7. Was Newborn Screening (also known as the 'heel prick' test) to detect homocystinuria performed at birth for you or your child?
   - o Yes
   - o No
   - o Unsure

8. Were you or your child diagnosed as a result of Newborn Screening?
   - o Yes
   - o No

9. At what age did you or your child receive the definite diagnosis?
Age ______________________

10. If homocystinuria was missed by Newborn Screening (NBS), please indicate what year the NBS was performed and where it was conducted (including country, and where applicable, state or province)? ____

11. What was the total homocysteine (Hcy) blood concentration measurement (µmol/L) prior to commencement of treatment? ____

12. If you experienced/observed any of the following symptoms **prior to diagnosis** please record the age the symptom was experienced/observed by you and/or medical staff.

	Months	Years
Developmental delay		
Intellectual deficit		
Seizures		
Clumsiness		
Anxiety		
Depression		
Obsessive-compulsive disorder		
Behavioural disorder		
Personality disorder		
Learning difficulties		
Osteoporosis		
Scoliosis		
Abnormally protruding or sunken chest		
Blood clot(s)		
Strokes		
Pulmonary embolism		
Near sightedness (myopia)		
Lens dislocation		
Other		

Please detail any other symptoms not listed above and age (months and years) observed.

13. How long after first seeking medical advice for your/your child's symptoms was the possibility of a diagnosis of homocystinuria discussed with you by medical staff?
Years ____

14. How many doctors did you or your child see about symptoms/signs related to homocystinuria before receiving a confirmed diagnosis?

- o 1-2 doctors
- o 3-4 doctors
- o 5-6 doctors
- o 7-8 doctors
- o 9-10 doctors
- o 11 or more doctors

15. Which specialist (for example GP, Ophthalmologist, Metabolic specialist, etc) initially raised the possibility of the diagnosis of homocystinuria?___________________________________

16. Before a confirmed diagnosis was made were you incorrectly given another explanation for your symptoms?

- o Yes
- o No

Please describe in full details.____

17. Would you describe the process of getting a diagnosis difficult in comparison, for example, to a family member or friend obtaining a diagnosis of a DIFFERENT severe disease (e.g. cancer)?

- o Not at all
- o A little
- o Somewhat
- o Very
- o Extremely

18. If you believe the diagnosis was delayed please describe the consequences for your family?_____________________________________

19. How was the confirmed diagnosis communicated?

- o In person
- o By telephone
- o By letter
- o Other

If 'other' please specify. ____

20. What do you perceive as the reasons for the delay in diagnosis? Tick as many boxes as you feel relevant.

- Lack of knowledge about the disease amongst General Practitioners (GP)
- Lack of knowledge about the disease amongst specialists
- Lack of symptom awareness by family
- Delays in obtaining test results
- Lack of access to appropriate tests
- Long waiting times to see medical specialists

Other (please specify) ____

21. How do you feel about the way the diagnosis was delivered?

- Very satisfied
- Satisfied
- Somewhat satisfied
- Neither satisfied nor dissatisfied
- Somewhat dissatisfied
- Dissatisfied
- Very dissatisfied

Please describe why you felt this way. ____

22. Please provide any additional comments regarding diagnosis ____________________________

23. Were you offered psychological support?

- Yes
- No

Use of support services

24. Do you believe you have been provided with adequate information about the disease?

- Yes
- No

If you answered 'No' please describe what you would like. ___________________________________

25. Are you interested in being kept informed of current research and clinical trials related to the disease?

- Yes
- No

26. Was information on available support groups provided at diagnosis?

- Yes
- No

27. How interested are you in finding and utilising support groups and organisations?
- o Extremely interested
- o Very interested
- o Somewhat interested
- o Not so interested
- o Not at all interested

28. Have you found a relevant group in your country?
- o Yes
- o No

29. Have you searched for a support group internationally?
- o Yes
- o No

Impact of treatment

30. Please tick each treatment, if any, that applies to you or your child.
- o Low protein diet
- o Amino acid mixture
- o Betaine
- o Intramuscular injections of vitamins (such as hydroxocobalamin, cyanocobalamin or dominic acid)
- o Oral vitamins
- o Not on treatment

31. What is your daily allowance/daily target of protein/Met per kg body weight? ________________

32. What was your most recent total homocysteine (Hcy) blood concentration measurement (µmol/L)? __________
Please SPECIFY whether this measurement is dry blood spot or venous blood. __________

33. Are you or your child satisfied with the current treatment regime including low protein diet, amino acid mixture/specialised formula and/or vitamins?
- o Not at all
- o A little
- o Somewhat
- o Very
- o Extremely

Amino acid mixture/specialised formula

Please note the following questions apply to amino acid mixture/specialised formula ONLY.

34. In the past 7 days, did you argue with your child because of their amino acid mixture/specialised formula?

- o Never
- o A little of the time
- o Sometimes
- o Most of the time
- o Always
- o Not applicable

If so, why? ____

35. In the past 7 days, was it hard for you or your child to take the amino acid mixture/specialised formula?

- o Never
- o A little of the time
- o Sometimes
- o Most of the time
- o Always
- o Not applicable

If so, why? ____

36. In the past 7 days, was it hard for you or your child to do any particular activity because of the amino acid mixture/specialised formula? This may include attending a social event, party, sporting activity, etc.

- o Never
- o A little of the time
- o Sometimes
- o Most of the time
- o Always
- o Not applicable

37. In the past 7 days, did you or your child miss taking some amino acid mixture/specialised formula?

- o Never
- o 1 or 2 times
- o 3 to 5 times
- o 6 to 7 times
- o More than 7 times
- o Not applicable

If so, why? ____

Medications/vitamins (oral and intramuscular injections)

Please note the following questions apply to medications and vitamins (oral and intramuscular injections) ONLY.

38. In the past 7 days, was it hard for you or your child to take medication when supposed to? Medication includes oral vitamins and intramuscular injections but excludes formula.

- o Never
- o A little of the time
- o Sometimes
- o Most of the times
- o Always
- o Not applicable

If so, why? ____

39. Do you experience any difficulties accessing (i.e availability or affordability) your medication and/or vitamins?

- o Yes
- o No

If 'yes', please describe. ____

40. If you can, please estimate additional cost of medications/vitamins to your family PER MONTH. Please state what currency for example AUD, Euro, USD, etc. _________________________________

Low protein diet

Please note the following questions apply to the low protein diet ONLY.

41. In the past 7 days, was it hard for you or your child to follow the low protein diet?
- o Never
- o A little of the time
- o Sometimes
- o Most of the time
- o Always
- o Not applicable

42. In the past 7 days, did you or your child feel unhappy when diet was restricted because of homocystinuria?
- o Never
- o A little of the time
- o Sometimes
- o Most of the time
- o Always
- o Not applicable

43. In the past 7 days, was it annoying or challenging to weigh, measure or estimate protein in food?
- o Never
- o A little of the time
- o Sometimes
- o Most of the times
- o Always
- o Not applicable

44. In the past 7 days, was it time consuming to prepare low protein meals (weighing, measuring, cooking)?
- o Never
- o A little of the time
- o Sometimes
- o Most of the times
- o Always
- o Not applicable

45. In the past 7 days, did you or your child want to eat things others could eat?
- o Never
- o A little of the time
- o Sometimes
- o Most of the times
- o Always
- o Not applicable

46. In the past 7 days, was it hard for you or your child to do any particular activity because of the low protein diet? This may include attending a social event, party, sporting activity, etc.

- Never
- A little of the time
- Sometimes
- Most of the times
- Always
- Not applicable

47. In the past 7 days, did you or your child follow the low protein diet as directed?

- Never
- A little of the time
- Sometimes
- Most of the times
- Always
- Not applicable

48. In the past 7 days, while following the low protein diet did you or your child still enjoy eating?

- Never
- A little of the time
- Sometimes
- Most of the times
- Always
- Not applicable

49. In the past 7 days, did you argue with your child because of the low protein diet?

- Never
- A little of the time
- Sometimes
- Most of the times
- Always
- Not applicable

50. Do you have access to specialised low protein food products?

- Yes
- No

If 'yes' please describe any financial assistance you receive for the purchase of low protein foods.

51. If you can, please estimate additional cost of purchasing specialised low protein foods PER MONTH. Please state what currency for example AUD, Euro, USD, etc. . ________________________

52. Please provide any additional comments regarding the management and treatment of the disorder. ____

Use of health services

53. Do you or your child attend a IEM/metabolic specialty clinic?
   - Yes
   - No

54. Are you satisfied with your medical care?
   - Yes
   - No

55. Which health professional manages homocystinuria care for you or the person you care for? This may include ordering blood tests and managing referrals.
   - I do not have a health professional who manages my homocystinuria
   - General Practitioner (GP)
   - Paediatrician
   - Metabolic specialist
   - Neurologist
   - Geneticist
   - Haematologist
   - other

If you answered other please specify.____

56. Which word best describes how you would rate your medical care.
   - Poor
   - Fair
   - Average
   - Good
   - Excellent

57. How easy is it to talk to medical staff at your clinic about the disorder and any medical concerns?
   - Very easy
   - Easy
   - Somewhat easy
   - Neither easy nor difficult
   - Somewhat difficult
   - Difficult
   - Very difficult

58. In the past 12 months, if you contacted your clinic outside of appointments how often did you get an answer to your medical question as soon as you needed?
   - Always
   - Usually
   - Sometimes
   - Rarely
   - Never

Final comments

59. Do you have any further comments, questions, or concerns? ________________________
